# Intermittent hypoxic training – derived exosomes in stroke rehabilitation

**DOI:** 10.3389/fnint.2024.1475234

**Published:** 2024-09-11

**Authors:** Myoung-Gwi Ryou, Summer Burton

**Affiliations:** Department of Medical Laboratory Sciences, Public Health, and Nutrition Science, College of Health Science, Tarleton State University, Fort Worth, TX, United States

**Keywords:** exosome, stroke, hypoxia, intermittent hypoxic training (IHT), microglia, Neuroprotection

## Abstract

Ischemic stroke is the fourth leading cause of adult disability in the US, and it is a huge social burden all over the world. However, the efficient treatment of ischemic stroke is not available. An apparent reason for failing to find or develop an intervention for ischemic stroke is contributed to the tight blood–brain barrier (BBB). The unique characteristics of exosomes that can traverse BBB have been highlighted among researchers investigating interventions for ischemic stroke conditions. Additionally, intermittent hypoxic training has been considered a potential intervention in the treatment or rehabilitation process of ischemic stroke patients. In this mini-review, we are going to review the possibility of applying exosomes produced by a subject who does intermittent hypoxic conditioning in a treatment program for ischemic stroke.

## Introduction

In 2019, 77.19 million people worldwide had an ischemic stroke, which caused 11.6% of all deaths, making it the second leading cause of death and the top cause of chronic disability (World Health Organization, 2020; Pu et al., 2023). In the USA, it remained the fifth most common cause of death (Tsao et al., 2022). Stroke survivors often face lifelong disabilities that diminish their quality of life (Jiang et al., 2022; Gualerzi et al., 2021).

Cerebral ischemia causes reactive oxygen species accumulation, resulting in cerebral cell death and neuronal injury, with stroke pathophysiology being highly complex due to various endogenous and exogenous factors (Jung and Mallet, 2018; Mallet et al., 2018). The involvement of the immune response, excess inflammation, and programmed cell death in ischemic brain damage has been reported. BBB breakdown allows danger-associated molecular patterns (DAMP) and cytokines to leak into circulation, triggering systemic immunity, immunodepression, and severe infections. This can also cause cerebral edema due to vasodilation, increased BBB permeability, and cell swelling from ionic imbalance and inadequate metabolism (Gualerzi et al., 2021). Inflammation in the brain persists for years after stroke, which hinders long-term recovery and may contribute to the 5-year recurrence rate of 33% and mortality rate of approximately 70%.

Current stroke therapies, such as intravenous thrombolysis and endovascular thrombectomies, are effective but have a narrow therapeutic window. Delayed thrombolytic treatment can cause severe side effects, including potentially life-threatening hemorrhage (Ryou et al., 2013). Most of proposed alternative treatments for current limited options have failed clinical trials (Table 1) (Di Cesare et al., 2016; Elkind et al., 2020; Hill et al., 2020). Hence, additional therapeutic strategies are needed.

**Table 1:** Clinical trials of interventions for ischemic stroke.

Treatment	Clinical Trial Identifier	Trial type	Result	Reference
PF-03049423	NCT01208233	Pfizer Phase 2	No statistical significance between groups in the clinical trial	ClinicalTrials.gov (2016)
Natalizuma	NCT02730455	Phase 2b	No evidence of benefit	Elkind et al. (2020)
Nerinetide	NCT02930018	Phase 3	Did not improve the proportion of patients achieving good clinical outcomes compared to placebo	Hill et al. (2020)

Therefore, understanding ischemic stroke pathogenesis is crucial for developing treatments. Efficiently delivering anti-inflammatory and protective agents to the brain without worsening BBB integrity could improve patient outcomes. This review explores the potential of exosomes obtained from an intervention in treatment and rehabilitation of stroke.

## Methods

This review summarizes concepts and evaluates results from several research articles on related topics. It was conducted using the electronic literature databases PubMed, Academic Search Complete (EBSCO), and BioOne Complete. We narrowed down the academic and professional publishers available by searching the following keywords: exosomes, intermittent hypoxic training, ischemic stroke, and hypoxia. The search was performed by prioritizing the newest material to evaluate recent progress in this field, but there were no strict restrictions on dates if the content was relevant.

### Exosomes

Exosomes are microvesicles (30–150 nm) in diameter, have a density of 1.08–1.19 g/mL, and can be found in most physiological fluids (Sidhom et al., 2020). Exosomes are secreted from diverse cells and are coated with a phospholipid bilayer derived from the membrane of the cell of origin. Upon being released into the extracellular medium, exosomes interact with the target cells located systemically or nearby and are involved in humoral intercellular communication. The unique characteristic is that exosomes contain cell-specific cargos of proteins, lipids, DNA, mRNA, and microRNA(miR). Hence, an intervention that can manipulate the biologically important cargo molecule carried by exosomes makes them a potential target for novel therapeutic manipulation and diagnostic biomarkers.

**Figure 1:**
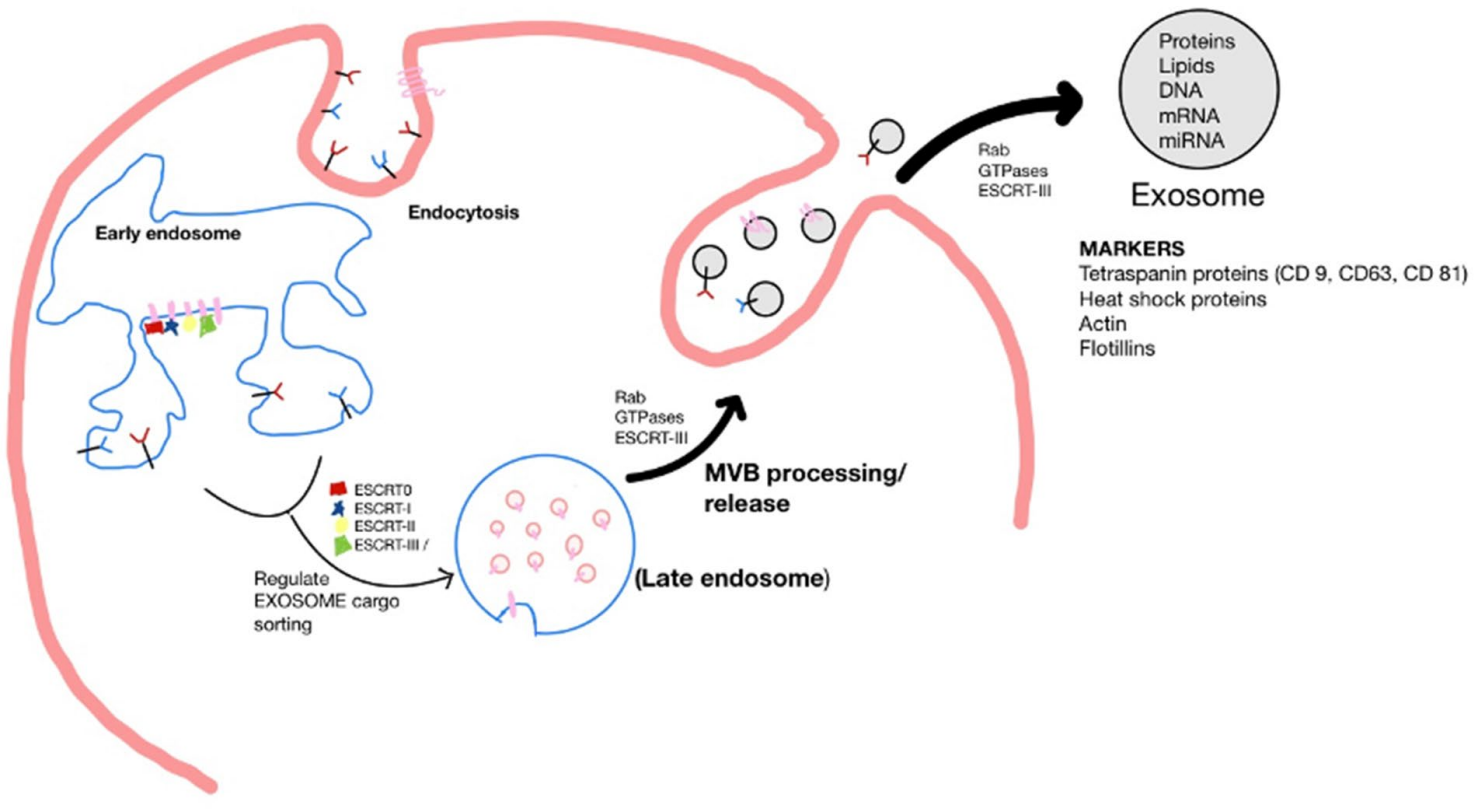
Schematic of the biogenesis of the exosome. Where, Endosomal-sorting complex required for transport (ESCRT); Multivesicular body (MVB).

Exosomes originate by the endocytosis pathway in a cell of origin as vesicles known as multivesicular bodies (MVBs). The endosomal-sorting complex required for transport (ESCRT) family orchestrates early exosome’s molecular composition and formation. For example, ESCRT-0, ESCRT-I, and ESCRT-II regulate exosome cargo sorting. MVBs processed by lysosomes fuse with the plasma membrane to be released as mature exosomes. The secretion of exosomes is controlled by signal transduction factors, such as Rab GTPases and ESCRT-III, which undertake the deformation and fission of the membrane (Jiang et al., 2022; Cun et al., 2022). Upon being secreted, exosomes can be detected by many markers, including the tetraspanin family proteins (CD9, CD63, and CD81), heat shock proteins (Hsp), actin and flotillins, an endosomal sorting complex required for transport proteins (Alix and TSG101) and integrins (Jankovičová et al., 2020). However, the composition profile of exosomes is greatly influenced by the type and state of parental cells (Figure 1) (Ferreira et al., 2022).

### Biocompatibility of exosomes

Exosomes are unique in representing the molecular signature of their particular cell-of-origin and can cross most anatomical barriers, including BBB, due to their biocompatibility (Ferreira et al., 2022). Therefore, exosomes can be exploited to reach hard-to-reach organs and to aid delivery to specific target sites (Rech et al., 2022). Exosomes can be retrieved and administered by minimally invasive procedures. Multiple techniques have been used for exosome isolation, including differential centrifugation (Kamerkar et al., 2017), ultrafiltration (Klymiuk et al., 2019), and size exclusion chromatography (Stranska et al., 2018).

Through surface engineering strategies, targets of exosomes and their circulation time can be modified accordingly, which can be achieved by sonication (Pham et al., 2021; Zhu et al., 2022), electroporation (Jia et al., 2018; Haney et al., 2020), and incubation (Belhadj et al., 2020). Furthermore, isolated exosomes can be loaded with therapeutic cargo, including small molecular-weight drugs, nucleic acids, proteins, and viral vectors (Ferreira et al., 2022). The most straightforward technique to enrich them with modifying agents is incubation (Gong et al., 2019). This reasoning explains the surge in research aimed at improving therapy outcomes and focusing on precision medicine through exosome therapy (Gualerzi et al., 2021).

### Clinical application of exosomes

Exosomes’ diverse applications span diagnostic procedures, where they serve as carriers of biomarkers essential for disease detection and monitoring. Stem cell-derived exosomes are particularly valuable in facilitating tissue repair and regeneration. Emerging data suggests exosomes manifest several favorable features in their utilization as endogenous drug delivery in many therapies due to their low immunogenicity, innate stability, and high delivery efficiency. For example, engineered exosomes were exploited to simultaneously deliver an anticancer drug 5-FU and miR-21 inhibitor oligonucleotide (miR-21i) to Her2-expressing cancer cells. Tumor-bearing mice indicated a significant anti-tumor effect by inducing cell cycle arrest, reducing tumor proliferation, and increasing apoptosis (Liang et al., 2020).

Furthermore, they have been studied to aid therapy effects in a wide variety of other prevalent diseases worldwide, including different cancers such as colon and non-small cell lung cancer (NSCLC), myocardial infarction, kidney failure, and wound healing (Liang et al., 2020; Hu et al., 2023; Zhu et al., 2018; Kim et al., 2021; Shi et al., 2021). Exosomes have also been manipulated to target the brain specifically by surface modifications. The utilization of iron oxide nanoparticles (IONP) on the surfaces of exosomes not only enhances cell targeting but upregulates the expressions of angiogenic factors (Ang-1, FGF2, and VEGF) and anti-inflammation factors (TGF-β1 and TGF-β3) (Kim et al., 2020).

Exosomes are favorable in treating central nervous system (CNS) disorders because of their unique properties, as described earlier, and facilitate intercellular communication in the intracranial environment (Rech et al., 2022). Exosomes mediate therapeutic effects by transferring their cargos, thus modulating various pathways; all these types of cargo can result in multiple cerebroprotective properties in ischemic conditions, including angiogenesis, neurogenesis, anti-apoptosis, and inflammation (Jiang et al., 2022). Therefore, utilizing these exosomes to treat pathological conditions of the CNS is being focused.

The cargo potential of exosomes has been explored specifically with microRNAs (miR) to develop an efficient delivery system (Gong et al., 2019). MicroRNAs have critical roles in regulating gene expression and hold great promise for enhancing the efficiency of ischemic stroke treatment, given their ability to regulate multiple neuroprotective-targeted genes and related pathways (Tian et al., 2021). A study focused on the master hypoxia-induced miR-210, which promotes angiogenesis mediated by the vascular endothelial growth factor (VEGF) signaling pathway. Mice were subjected to 60 min of middle cerebral artery occlusion and 24 h of reperfusion (MCAO/R) to mimic an ischemic stroke, and, after 24 h of reperfusion, engineered exosomes carrying miR-210 were intravenously administered. After 14 days post-treatment, angiogenesis was improved, and there was a significant increase in animal survival (Zhang et al., 2019).

Furthermore, exosome-based intervention also improves neurogenesis by enhancing the differentiation of stem cells. The exosome enriched with MiR-17-92 cluster harvested from MSCs significantly improved neurological function and enhancements of oligodendrogenesis, neurogenesis, and neurite remodeling. It is suggested that this is due to targeting PTEN to activate the PI3K/Akt/mTOR, which leads to cell proliferation (Xin et al., 2017). Enkephalin delivery via exosomes released from MSCs is successful in preventing apoptosis. Enkephalin is an endogenous opioid peptide that decreases the expression of p53, caspase 3, and NO, contributing to apoptosis. At the same time, it increased neuronal density and accelerated neurological recovery after stroke was induced in rat models (Liu et al., 2019). Exosomal miR-137 was upregulated and participated in the partial neuroprotective effect by the Notch1 pathway (Zhang D. et al., 2021). Exosomes loaded with curcumin, known as antioxidant and anti-inflammatory, have strongly suppressed the lesion’s inflammatory response and cellular apoptosis (Tian et al., 2018; Hewlings and Kalman, 2017). Furthermore, Liu et al. reported that exosomes alleviated neuronal damage caused via pyroptosis via the inhibition of NLRP3 inflammasome-mediated inflammation; pyroptosis is a highly inflammatory mode of regulated cell death and contributes to the pathophysiology of ischemia–reperfusion (I/R) injury (Liu et al., 2021).

Exosomes released from microglial cells contribute to neuroprotection after stroke by anti-apoptotic and anti-inflammatory effects. Microglia, a resident cerebral phagocyte, is responsible for clearing metabolic wastes to maintain CNS homeostasis and promote removing or recovering brain cells injured by ischemia. Noxious conditions polarize the microglia to M1 (classical) or M2 (alternative) phenotypes. M1 microglia mediate pro-inflammatory responses, and M2 microglia play critical roles in healing and repairing damaged cells. M2 microglia-derived exosomes attenuated ischemic brain injury and promoted neuronal survival via exosomal miR-124 and its downstream target USP14, both antiinflammation regulators (Song et al., 2019). Furthermore, exosomes derived from M2 microglia attenuated neuronal apoptosis, decreased infarct volume, and reduced behavioral deficits in ischemic mice. Exosomes enriched with zinc finger E-box binding homeobox 2 protein (zeb2) and Axin2 have also enhanced neurogenesis. Zeb2 is a transcriptional regulator and is associated with the transforming growth factor β (TGF-β) signaling pathway, which is essential during early neurodevelopment. Axin 2 is related to TGF-β pathway, related to endothelial cell angiogenesis and Ent/ β-catenin pathway. These results show that the treated exosomes induced functional recovery after stroke by stimulating endogenous neurogenesis in MCAO rats, and the mechanism is believed to be related to the SOX10, Wnt/β-catenin, and endothelin-3/EDNRB pathways (Wei et al., 2022) (Table 2).

**Table 2:** Clinical application of exosomes.

Clinical application	Role of exosomes
Disease Detection and Monitoring	Exosomes serve as carriers of biomarkers, essential for diagnosing and monitoring various diseases.
Regenerative Medicine	Stem cell-derived exosomes are valuable in regenerative medicine, aiding in tissue repair and regeneration.
Drug Delivery	Engineered exosomes offer targeted and efficient transport of therapeutic agents. They are utilized as endogenous drug delivery systems in various therapies due to their low immunogenicity, stability, and high efficiency.
Cancer Therapy	Exosomes delivering anticancer drugs (e.g., 5-FU) and miRNA inhibitors (e.g., miR-21i) to specific cancer cells show significant anti-tumor effects, including cell cycle arrest, reduced tumor proliferation, and increased apoptosis.
Central Nervous System Disorders	Exosomes have intrinsic properties that enable them to cross the blood–brain barrier (BBB), making them favorable for treating CNS disorders. They facilitate intercellular communication in the brain and mediate therapeutic effects by transferring various protective cargos.
Stroke and Ischemic Conditions	Exosome-based therapies have shown potential in stroke treatment by promoting angiogenesis, neurogenesis, anti-apoptosis, and reducing inflammation.
Anti-inflammatory and Anti-apoptotic Effects	Exosomes can be loaded with therapeutic agents such as curcumin or microRNAs to reduce inflammation and cellular apoptosis in various conditions, including ischemic brain injury.
Neurogenesis and Neuroprotection	Exosomes derived from microglia or stem cells can enhance neurogenesis, promote neuronal survival, and contribute to functional recovery after stroke. They achieve this by carrying specific molecules (e.g., miR-124, zeb2, Axin2) that regulate key signaling pathways involved in brain repair and neuroprotection.

Exosomes could be used as biomolecule carriers to enhance interventions’ outcomes. Endogenous exosomes harvested from specific treatments effectively deliver endogenous protective agents to the damaged area.

### Clinical applications of intermittent hypoxic training in stroke

Hypoxic stresses can elicit a series of physiological responses in our system and are involved in many physiological and pathological processes (Yang et al., 2023). Stroke recovery is associated with restorative events that increase angiogenesis and neurogenesis levels and reduce inflammation.

Intermittent hypoxia (IH) at approximately 10% oxygen level has been documented to improve ischemic injury in cerebrovascular diseases and associated risk factors (Yuan et al., 2022; Jackman et al., 2014). For example, short-term IHT has been shown to promote gliogenesis in middle cerebral artery occlusion rat models (Roshan et al., 2023). IHT has also been shown to promote the recovery of motor function of cerebral ischemia by regulating mitochondrial function (Su et al., 2022). It has been reported that Repetitive hypoxic preconditioning has been shown to upregulate the chemokine CXCL12, which contributes to the endogenous, anti-inflammatory phenotype before stroke onset (Selvaraj et al., 2017).

Based on favorable reports, IHT could be used as an intervention for ischemic stroke therapy. Recently, we reported that nIHT modulates microglial polarization, specifically increasing the rate of M2 phenotype (Tantingco and Ryou, 2020). Microglial phenotype should be considered when developing interventions for ischemic stroke patients due to its anti-inflammatory and neuroprotective properties. Overall, evidence suggests nIHT may be an innovative, non-invasive way to augment damage and promote recovery associated with neurovascular injury.

Challenges regarding IHT have been documented. Despite being noninvasive and nonpharmacological, it requires intense investigation for safe application to patients in a clinical setting. The brain’s metabolism is highly active and very sensitive to changes in oxygen levels, especially in several groups of individuals. Elderly patients who are more at risk for IS are also susceptible to hypoxia due to preexisting heart or lung conditions.

Therefore, the intensity and timing are essential and may vary significantly between patient groups. Since this is also a systemic and multi-targeted intervention, off-target effects and potential damage to other organs must be explored (Yuan et al., 2022).

### Clinical applications of pre-conditioned exosomes

It is well documented that hypoxic conditions affect exosome contents, thereby regulating various functions that may promote or augment pathophysiology. Exosomes pre-conditioned with hypoxia *in vivo* have recently been explored in one study where they determined that the expression of exosomal metabolites and proteomics varied significantly compared to controls (Fan et al., 2023). Similarly, these results have been reinforced *in vitro* by next-generation sequencing, where exosomal-miRNAs expression profile and function were differentially expressed between hypoxic preconditioned and control groups (Zhang et al., 2018). Specific cell lineages and the duration and severity they are exposed to hypoxia exhibit divergent exosome changes (Senda et al., 2023). Nevertheless, the exact mechanisms underlying the hypoxia regulation on exosome production, cargo composition, and function still need to be understood entirely.

Exosomes that have been pre-conditioned with hypoxia are the current focus of research. Investigating changes in exosomes and their role in hypoxic injury and adaptation may help in preventing secondary damage caused by hypoxia. It has been widely reported that these conditioned exosomes obtained after hypoxia may have therapeutic potential in various diseases, including ischemic-related injury. For example, exosomes extracted from splenic ischemic preconditioning models exerted a protective effect to attenuate renal I/R injury by reducing apoptosis and increasing TNF-α and interleukin-1β (Liu and Shen, 2023). In addition, repeated remote ischemic conditioning is cardioprotective in rat models after myocardial infarction (Luo et al., 2023; Yamaguchi et al., 2015).

Furthermore, novel studies have investigated the effects of preconditioned exosomes on the central nervous system. Exosomes secreted from MSCs under ischemia carry an increased concentration of miRNA-21, which may be responsible for mediating synaptic dysfunction and regulating inflammatory responses in APP/PS1 mice. Rescuing cognitive decline is an attractive approach to developing a potential treatment for Alzheimer’s disease (Cui et al., 2018).

Neuronal death is a leading cause of the pathophysiology following spinal-cord injury (SCI). Therapies that target revascularization and inflammation are vital components when exploring treatment options for spinal cord repair. Exosomes derived from hypoxia preconditioned human umbilical vein endothelial cells have demonstrated angiogenic function by cell-to-cell communication with mesenchymal stem cells (MSCs) in spinal cord repair. Stimulated MSCs showed significant tube formation within 2 h and promoted nerve tissue repair following spinal cord transection in rats through their proangiogenic and anti-inflammatory effects (Li et al., 2022) Additionally, exosomes derived from hypoxia-conditioned adipose tissue-derived stromal cells significantly reduced neuronal apoptosis after OGD in rat SCI models; miR-499a-5p expression in these exosomes was believed to regulate the JNK3/c-jun-apoptotic signaling pathway by targeting JNK3 (Liang et al., 2022). Another study noted miR-216a-5p shuttled by pre-conditioned exosomes helps repair traumatic spinal cord injury by shifting microglial M1/M2 polarization (Liu et al., 2020). These results combined all support pre-conditioned exosome therapy to reduce neuronal damage after spinal cord injury and may pose an effective treatment strategy.

### Clinical applications of pre-conditioned exosomes in stroke

Pre-conditioned exosomes derived from human microglial cells, astrocytes, adipose-derived stromal cells, and neural and mesenchymal stem cells have therapeutic effects on neuronal injury *in vitro* (Zhang D. et al., 2021; Zhang L. et al., 2021; Xin et al., 2023; Yang H. et al., 2022; Ye et al., 2022; Guitart et al., 2016).

Cerebral ischemia induces inflammation due to high levels of cytokines and chemokines at the early stage of the disease. Extracellular vesicles (EV) from hypoxia-preconditioned microglia promote tissue regeneration and neurological recovery in stroke mice via the TGF-β/Smad2/3 pathway. This is due to the M2 phenotype shift post-conditioning, which yields enrichment of the TGF-β1 protein inside EVs harvested from microglia. These EVs cross the BBB to further increase the numbers of resident anti-inflammatory M2 microglia within the ischemic brain. M2 microglia alter the cellular microenvironment following cerebral damage by clearing cell debris and releasing immunomodulatory factors.

Furthermore, TGF-β1 activates the Smad2/3 signaling pathway, ultimately contributing to cellular protection and increased neurological recovery both *in vitro* and *in vivo* (Zhang D. et al., 2021; Zhang L. et al., 2021). In addition, another study confirmed that preconditioned EVs from microglia augmented M2 polarization and repressed M1 microglia polarization even further than non-conditioned EVs. They report a decreased periinfarct AQP4 depolarization, brain edema, astrogliosis, and inflammation in stroke mice (Xin et al., 2023).

Furthermore, previous investigations have shown therapeutic effects of using hypoxic pre-treated adipose-derived stromal cell exosomes in cerebral ischemic injury. The mechanism involves improved cognitive function after cerebral infarction via delivery of circ-Rps5; circ-Rps5-mediated SIRT7 suppresses LPS-induced inflammation and apoptosis through the NF-κB signaling pathway. Neuronal damage was attenuated, and the hippocampus’s microglia shifted from M1 to M2 phenotype (Yang H. et al., 2022; Yang C. et al., 2022).

Vascular remodeling is essential for a better prognosis after a stroke. Infarct-preconditioned exosomes of umbilical cord mesenchymal stem cells promoted neurological recovery after stroke in rats by minimizing apoptosis and enhancing migration and vascular endothelial remodeling (Ye et al., 2022). Another study suggested that hypoxia enhanced the angiogenic potential of DPSC-derived exosomes via the transfer of lysyl oxidase-like 2 (LOXL2) (Li et al., 2023).

Hypoxic pre-conditioned exosomes isolated from neural stem cells SCs (NSCs-Ex) have shown neuroprotective characteristics. NSCs-Ex prevents cerebral injury by transferring miR-150-3p, which promotes neuron proliferation and suppresses neuronal apoptosis by inhibiting the CASP2 signaling pathway *in vitro* (Luo et al., 2022). These results have been reconfirmed in another study *in vivo* preconditioning on NSCs exosomes further exerted therapeutic effects on both survival and behavioral outcomes in ischemic stroke mice. Overall, hypoxic preconditioning NSCs can produce effective nano agents and may represent a promising strategy for clinical neurorestorative therapy (Jiang et al., 2023).

Exosomes derived from astrocytes under oxygen/glucose deprivation conditions were said to increase prion protein (PrP) levels. PrP has a protective role for neural cells, specifically against ischemic injury (Onodera et al., 2014).

Future studies could, therefore, investigate the neuroprotective adaptations of exosomes after repetitive low-dose nIH exposures and how this may be used to advantage in an innovative new strategy for stroke patient rehabilitation.

## Conclusion

The brain, which consumes the most oxygen of any organ, is highly sensitive to damage that may be induced by hypoxia, which is closely related to stroke and other brain diseases. However, nIHT has shown promising effects to mitigate harmful effects and enhance protective responses to protect the brain from ischemic injury. As an important intercellular player in neurovascular communication, the exosome mediates cerebral injury by transferring protein and RNA cargoes.

In conclusion, novel evidence suggests exosomes that have undergone hypoxic preconditioning have neuroprotective properties, but the therapeutic effects of intermittent hypoxia have not yet been explored. Published journals investigating exosomes that have been pre-conditioned with IHT are very limited. Nevertheless, exploring the potential benefits of combining IHT and exosome therapy would be of great value, which has been noted separately as successful. In contrast, several areas allow improvement of current strategies for disease treatment using exosomes. Current isolation and purification techniques are time-consuming and have low yield rates (Stranska et al., 2018). Challenges associated with the rapid biological clearance rate of exosomes post-local administration must be addressed to maximize therapeutic efficacy while minimizing unwanted effects.

Furthermore, IHT can elicit different exosome responses depending on their cell lineage and the hypoxic exposure’s duration, frequency, and intensity.

Overall, the protective mechanisms underlying intermittent hypoxic conditioning and utilizing exosomes are complex and diverse and have yet to be fully explored. Despite this, current evidence suggests this may hold great potential in stroke rehabilitation in the future.
